# Genome and transcriptomics provide insights on stipular spine morphogenesis in *Robinia pseudoacacia*

**DOI:** 10.48130/forres-0026-0003

**Published:** 2026-01-31

**Authors:** Yanting Tian, Ye Zhao, Yuhan Sun, Wenhao Bo, Xiong Huang, Jialong Wen, Shuzhi Wang, Yanping Jing, Yifan Zhao, Tianle Shi, Yousry A. El-Kassaby, Baozhen Zhang, Yuanshuai Zhang, Hao Yang, Zuodeng Peng, Juan Han, Yun Li, Quanzi Li

**Affiliations:** 1 State Key Laboratory of Tree Genetics and Breeding, Engineering Technology Research Center of Black Locust of National Forestry and Grassland Administration, National Engineering Research Center of Tree Breeding and Ecological Restoration, College of Biological Sciences and Technology, Beijing Forestry University, Beijing 100083, China; 2 College of Forestry, Sichuan Agricultural University, Chengdu, Sichuan 611130, China; 3 College of Materials Science and Technology, Beijing Forestry University, Beijing 100083, China; 4 Laboratory of Archaeological Sciences and Cultural Heritage Conservation, Chinese Academy of History, Beijing 102488, China; 5 Institute of Archaeology, Chinese Academy of Social Sciences, Beijing 100101, China; 6 School of History, University of Chinese Academy of Social Sciences, Beijing 102488, China; 7 Institute of Forestry and Pomology, Beijing Academy of Agriculture and Forestry Sciences, Beijing 100097, China; 8 Department of Forest and Conservation Sciences Faculty of Forestry, The University of British Columbia, 2424 Main Mall, Vancouver, BC V6T 1Z4, Canada; 9 State owned Daqingshan Forest Farm, Feixian County, Linyi, Shandong 273402, China; 10 Dulou Town People's Government, Xiao County, Suzhou, Anhui 235291, China; 11 The Key Laboratory for Silviculture and Conservation of Ministry of Education, College of Forestry, Beijing Forestry University, Beijing 100083, China; 12 National Key Laboratory for Development and Utilization of Forest Food Resources, Zhejiang Key Laboratory of Forest Genetics and Breeding, International Research Center for Plant Cell Wall, College of Forestry and Biotechnology, Zhejiang A & F University, Hangzhou, Zhejiang 311300, China

**Keywords:** Stipular spine, *Robinia pseudoacacia*, Genome, Transcriptomics

## Abstract

*Robinia pseudoacacia* (black locust) is a widely introduced and extensively cultivated species notably for its specialized thorn-like structure, known as the stipular spine. Here, combining Oxford Nanopore high-accuracy long-read sequencing and high-throughput chromatin conformation capture (Hi-C) scaffolding, a *de novo*, chromosome-level assembly of the *R. pseudoacacia* genome is presented, with a total size of 681.6 Mb and an N50 of 1.1 Mb. Observations showed that fiber cells beneath the epidermis of stipular spines undergo extensive lignification. The total lignin content is significantly higher in stipular spines (48.57%−63.71%) than in stem xylem (24.57%−27.99%), with syringyl (S-type) lignin being the predominant form in both, accounting for 69.79%−73.27% of the total lignin. By leveraging this genome, the transcriptomic and time-ordered gene co-expression network (TO-GCN) analyses uncovered core regulatory networks underlying stipular spine lignification, in which NAC transcription factors RopNST1/2 regulate secondary cell wall thickening and lignin biosynthesis during stipular spine hardening and the monolignol biosynthetic pathway enzyme genes *RopCCoAOMT3*, *RopHCT13b*/*79*/*48*/*14b*/*20*/*86*/*9a,* and *RopCCR3b*/*16*/*24*/*5a* act downstream during lignin biosynthesis. Collectively, these results provide cytological and molecular insights into the hardness of black locust stipular spines, and the high-quality reference genome offers a valuable resource for genomic and evolutionary studies in this species.

## Introduction

Thorns are needle-like metamorphosed structures formed in some plants, primarily serving as physical defenses to deter herbivores and other threats^[1]^. In addition to their protective role, prickles in roses also function in water storage^[2,3]^. Based on the origin, these structures are categorized into three main types: stem thorns (commonly referred to simply as thorns), as in citrus; leaf spines, found in cacti; and stipular spines, present in black locust; and prickles, characteristic of roses^[4−6]^. Some plants, such as thorn datura, cocklebur, and endive, possess fixed defensive structures with more complex developmental origins. Stipular spines are evolutionarily distinctive in their origin from leaf stipules, and their functional significance extends beyond mere defense. In species such as *Robinia pseudoacacia* L. (black locust), stipular spines play a key role in ecological adaptation^[7]^.

Cytological observations and characterizations of key regulators provide insights into spine formation. Stem thorns arise from axillary shoot apical meristems. In citrus, disruption of two TCP transcription factors (TFs), THORN IDENTITY1 (TI1) and TI2, leads to the conversion of thorns to branches^[8]^. Leaf spines are believed to develop from metamorphic leaves, while prickles are thought to originate from specialized epidermal hairs in the epidermis or cortex^[5,6,9]^. However, studies have shown that rose prickles originate from the subcutaneous meristem^[3]^. Despite these findings, knowledge about the molecular mechanisms underlying the origin and development of leaf/stipular spines and prickles remains limited. In *Citrus*, loss of function of MYB TF SST1 (SHORT AND SOFT THORN 1), a direct upstream regulator of SNT1 and SND1 (master regulators of secondary wall thickening), leads to shorter stem thorns and reduced hardness, which results from the inhibition of secondary cell wall biosynthesis in thorns^[10]^. It is proposed that stem thorns and prickles share the conserved mechanism governing their hardening^[10]^. Whether leaf/stipular spines share the same molecular basis for their sclerosis needs further studies.

*R. pseudoacacia* is a species native to the United States. It has been widely introduced to many countries for cultivation due to its rapid growth, rigid wood, and high economic value^[11,12]^. Due to its strong adaptability to adverse climatic conditions, such as drought and low precipitation, as well as its ability to grow in hilly or desertified areas with poor or depleted soil, black locust has become one of the three most successfully introduced tree species worldwide^[11,13,14]^. Currently, the total global area under black locust cultivation has reached 1.89 million hectares, with 1.23 million hectares in China. The species has played a significant role in afforestation efforts for soil and water conservation, landscape greening, and ecological restoration. It is also widely used in economic forests, including fodder forests, honey-source forests, and timber forests^[15,16]^.

In this study, stipular spine formation is investigated. The hypothesis posits that the hardening of stipular spines may result from lignin accumulations in the secondary cell walls of subepidermal sclerenchyma cells, and that the secondary cell wall thickening in these cells is mediated by NAC TFs in a manner analogous to xylem cell wall thickening. Selecting *R. pseudoacacia* (a highly adaptable species with significant ecological and economic value) as the model plant for stipular spine research can address the long-standing gap in stipular spine molecular biology—an area lagging far behind stem thorn or leaf spine studies. Histological microscopy was combined with lignin content/composition analysis to examine lignin accumulation in stipular spines. To understand the lignin biosynthesis and regulation at the molecular level, a *de novo* genome assembly and annotation were first performed in the large-spined clonal line AGT. Using the genome as a reference, RNA-seq, whole-transcriptomic, and time-ordered gene co-expression network (TO-GCN) analyses were conducted. The findings provide an important resource that enhances understanding of the fundamental biology and molecular basis of stipular spine formation in *R. pseudoacacia*.

## Materials and methods

### Plant materials

Several large (AGT, LC85, LC179, LC223, MC815, and BL01) and small (LC, LC110, LC197, 820026, and LC63) stipular spines varieties of black locust (*R. pseudoacacia* L) were collected from the State-owned Daqingshan Forest Farm in Fei County, Linyi City, Shandong Province, and used in this study.

### Genome sequencing, assembly, and annotation

A single black locust tree (variety AGT) was used for *de novo* genome assembly (Supplementary Fig. S1). High-quality DNA was extracted from young leaves using a QIAGEN Genomic kit. DNA libraries were constructed and sequenced on PromethION and GridION X5 platforms, generating 95.5 Gb single-molecule real-time (SMRT) long-read data. The genome was assembled using SMART*denovo* (https://github.com/ruanjue/smartdenovo)^[17]^. The pre-assembled genome was polished by aligning the Illumina sequencing data to it using BWA with default parameters^[18]^, followed by three rounds of corrections with Pilon^[19]^. Redundans 0.13c^[20]^ was used to remove redundant sequences with a parameter of -identity 0.5 and -overlap 0.5. The BioNano clean data were used to further scaffold the Nanopore sketch genome^[21]^. SSR sequences in the genome were analyzed using the MISA software^[22]^. Repeat sequences were predicted by RepeatMasker (www.repeatmasker.org)^[23]^. In the evolutionary analysis, the OrthoMCL method^[24]^ was used to identify gene families, the Markov model^[25]^ was used to obtain the homologous gene family of each species, and the species phylogenetic tree was constructed using single-copy genes. During the species divergence time estimation process, time calibration was completed using Fourfold Degenerate Transversion (4DTV, which refers to transversions occurring at the fourfold degenerate sites of synonymous codons in DNA sequences and is a commonly used molecular marker in evolutionary research for estimating species divergence time) and the MCMCTREE software^[26,27]^. Meanwhile, analysis of gene family expansion and contraction was conducted for the gene families using the CAFE software^[28]^.

### Electron microscopy and histological section analysis

Stipular spines from the current-year branches of the AGT and LC varieties were collected, dehydrated, dried, and used for scanning electron microscopy (SEM) and transmission electron microscopy (TEM) observations, following the previously described method^[29]^. Embedded samples were sliced into 4 µm thick sections using a MICROM HM 35S microtome (Thermo Fisher Scientific, Waltham, MA, USA). Sections were dewaxed with xylene, stained with safranine *O*-fast green, and finally observed/photographed under an ECLIPSE E200 microscope (Nikon, Tokyo, Japan) equipped with an automatic photographic system.

### Gene family analysis

Hmmer and Blastp methods^[30]^ were combined to identify members of specific gene families in *R. pseudoacacia*. The target families included 11 gene families related to the monolignol biosynthetic pathway and the NAC gene family. The PFAM numbers used for identification included PF00221 (PAL), PF00067 (C4H/C3'H/F5H), PF00501 (4CL), PF02458 (HCT), PF00106 (CCR), PF00107 and PF08240 (CAD), PF18322 (CSE), PF01596 (CCoAOMT), PF00891 (COMT), and PF02365 (NAC). Protein sequences were aligned through MUSCLE^[31]^, and a maximum likelihood phylogenetic tree was constructed by RAxML^[32]^. The resulting tree was visualized using iTOL^[33]^.

### RNA-seq and whole-transcriptomic analysis

Different tissues were collected for RNA-seq. Leaflets were collected from current-year branches of the large-spined variety BL01 at three distinct developmental stages, including apical unexpanded leaflets, expanded young leaflets, and mature leaflets. Developing xylem and phloem tissues were scraped from the xylem and phloem side, respectively, of BL01 stems after the bark was peeled. Each pair of stipular spines on internodes 1–4 (IN1–4) was collected from AGT and LC110 varieties, respectively, representing four different developmental stages. RNA was extracted using the CTAB method^[34]^. RNA-seq libraries were prepared and sequenced on the Illumina HiSeq platform as previously described^[35]^. The raw data were filtered to obtain clean reads and then aligned to the *R. pseudoacacia* genome using HISAT2^[36]^. Differentially expressed genes (DEGs) were identified by DESeq2^[37]^.

Whole-transcriptome sequencing was performed on the stipular spines from four developmental stages, including stipular spines from IN1–3, IN5, IN7, and IN10 of AGT. RNA was extracted using TRIzol reagent (Invitrogen, USA). Small RNAs (18−30 nt) were isolated by 15% denaturing polyacrylamide gel electrophoresis (PAGE) and recovered via crush-and-soak elution in 0.3 M NaCl at 4 °C overnight. Libraries were constructed following the NEBNext protocol (Multiplex Small RNA Library Prep Set for Illumina, Cat. No. E7580). Small RNA species (miRNAs, piRNAs, tRFs) were annotated and quantified using miRDeep2^[38]^ and piRBase^[39]^. Differentially expressed genes were analyzed by DESeq2^[37]^.

### Lignin content determination in xylem and stipular spines

Lignin content was determined for stem xylem and mature stipular spines in four black locust varieties, with three independent measurements for each sample. Samples (AGT-Sx, AGT-Ss, LC-Sx, LC-Ss, LC110-Sx, LC110-Ss, LC179-Sx, and LC179-Ss) were ground to a fine powder, lyophilized, and then subjected to sequential extraction with a chloroform/methanol mixture (2:1, v/v), methanol, and water at ambient temperature to remove soluble extractives. The resulting cell wall residues were re-lyophilized. Lignin content was determined using the Klason method^[40]^, and lignin structures were analyzed by NMR^[41,42]^. SPSS 20.0 was employed to perform significant difference analysis.

### Time*-*ordered gene co*-*expression network (TO-GCN) analysis

The construction of TO-GCNs was conducted with gene expression matrices from four groups (RPSI, RPSII, RPSIII, and RPSIV) as inputs, following Chang et al.^[43]^. The gene expression levels are provided in the Supplementary Data 1. Pearson's correlation coefficient (PCC) values were calculated for each pair of genes. The PCC cutoff value of 0.86 was applied for interactions between TF, miRNA, and non-TF genes. With this threshold, a TO-GCN was constructed in the 'C1 + C2+' mode, following the established procedure. To generate hierarchical levels of TO-GCN, SBP TFs with consistent expression trends from RPSI to RPSIV were selected as seed genes for subsequent analysis.

## Results

### Genome assembly and annotation

**Figure 1:**
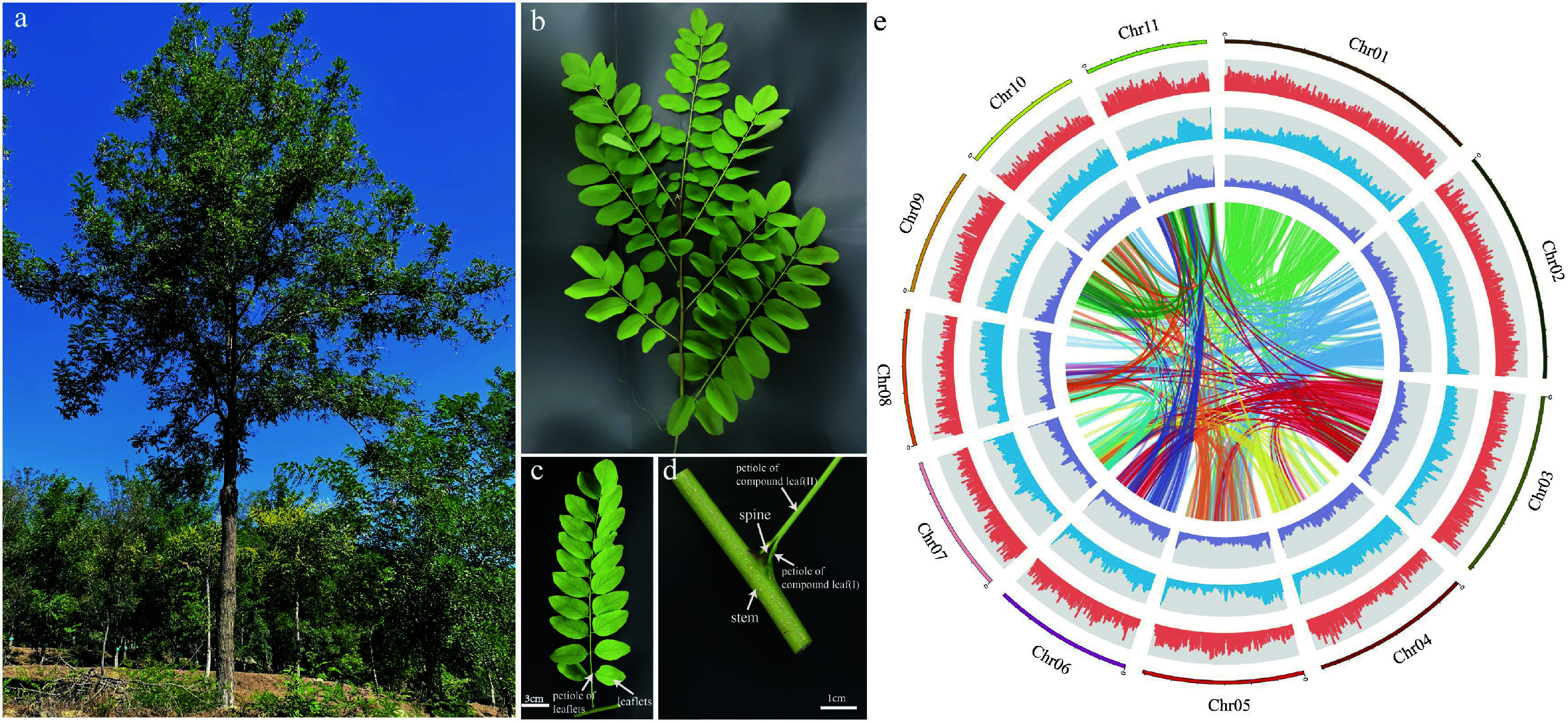
Plant morphology and genome features of *Robinia pseudoacacia*. (a) A *R. pseudoacacia* tree. (b) A stem branch. (c) A compound leaf. (d) Stipular spines of *R. pseudoacacia*. (e) The circle diagram shows the 11 chromosomes of *R. pseudoacacia* with a resolution of 1 Mb, gene density with a sliding window of 100 Kb, percentage of repeats with a sliding window of 100 Kb, GC content with a sliding window of 100 Kb, each linking line in the center of the circle connects a pair of homologous genes from inside to outside.

*De novo* genome assembly was conducted using 'AGT', an asexual line with stable inheritance of the large stipular spine trait (Fig. 1a−d). The estimated genome size was 663 Mb, and genome heterozygosity was 1.4% (Supplementary Fig. S2). The genome was assembled based on 95.55 and 109.7 Gb sequencing data from the Nanopore Sequel and Illumina HiSeq platforms, respectively. The assembled genome size was 681.6 Mb, with a contig N50 of 1.1 Mb, of which 681.29 Mb were anchored to 11 chromosomes, based on 48.3 Gb high-throughput chromatin conformation capture (Hi-C) data (Fig. 1e, Supplementary Tables S1, S2). To evaluate the assembled genome quality, Illumina and RNA-seq reads were first aligned to the genome, and the mapping rates were 98.38% and 89.43%, respectively. Further Benchmarking Universal Single-Copy Orthologs (BUSCO) evaluation showed that 97.1% of the complete gene models were covered (Supplementary Table S3, Supplementary Fig. S3a), and assessment of the completeness of the genome assembly by Core Eukaryotic Genes Mapping Approach (CEGMA) showed a 97.58% coverage of the conserved core eukaryotic genes^[44]^ (Supplementary Fig. S3b).

The annotation showed that a total of 359.4 Mb were repetitive sequences, representing 52.93% of the genome, with the retrotransposon (323.95 Mb) being the main transposon (TEs). Within the long terminal repeat (LTR) family, Gypsy and Copia families were predominant, accounting for 9.93% and 14.36% of the genome, respectively (Supplementary Table S4). A total of 40,605 genes were predicted from the genome by annotation (*de novo*, homology, and EST/cDNA sequence alignment), with an average gene length of 5,150.87 bp, average transcript length of 1,235.55 bp, and average exon and intron length of 279.96 bp and 1,147.06 bp. The BUSCO prediction analysis showed that about 92.2% of the complete gene elements could be found in the gene set, showing that the vast majority of the predicted genes were complete (Supplementary Table S3).

### Genome and gene family evolution

**Figure 2:**
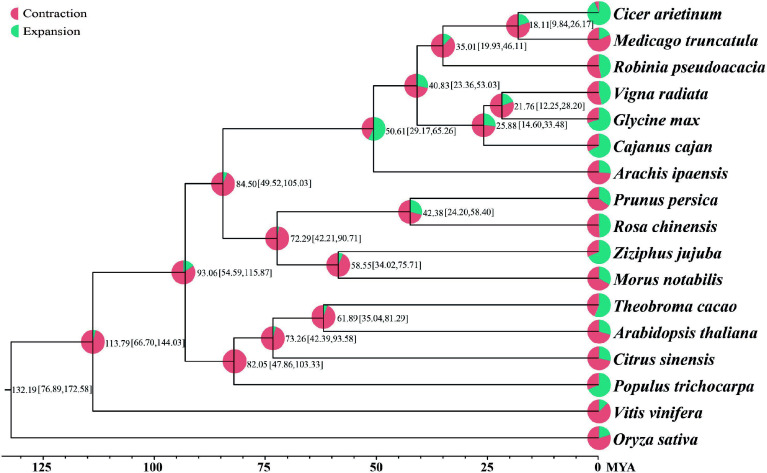
Phylogenetic tree of 17 plant species. Divergence time (MYA) estimations are indicated by the numbers.

Using the OrthoMCL method, 37,505 gene families containing 27,125 genes with the longest transcripts from 17 species (*Vitis vinifera*, *Populus trichocarpa*, *Citrus sinensis*, *Theobroma cacao*, *Arabidopsis thaliana*, *Arachis ipaensis*, *Cicer arietinum*, *Medicago truncatula*, *R. pseudoacacia*, *Cajanus cajan*, *Glycine max*, *Vigna radiata*, *Ziziphus jujuba*, *Morus notabilis*, *Prunus persica*, *Rosa chinensis*, *Oryza sativa*) were constructed. A phylogenetic tree of the species was built based on gene family data of 17 species. Divergence times were then estimated using Fourfold Degenerate Transversion (4DTV) sites and the MCMCTREE software. Finally, gene family expansion and contraction analysis was performed with the CAFE program. The phylogenetic tree indicated that 5,920 and 2,897 gene families underwent expansion and contraction, respectively, at the node of the species' evolutionary branch, which was dated to approximately 35.01 million years ago (MYA) (Fig. 2, Supplementary Data 2). Further Gene Ontology (GO) enrichment analysis revealed that the expanded genes were annotated to processes such as regulation of biological process, biological regulation, and regulation of nucleic acid-templated transcription (Supplementary Fig. S4). The contracted genes were annotated to the processes including defense response by cell wall thickening, defense response by callose deposition in cell wall, and cell wall modification (Supplementary Fig. S5).

**Figure 3:**
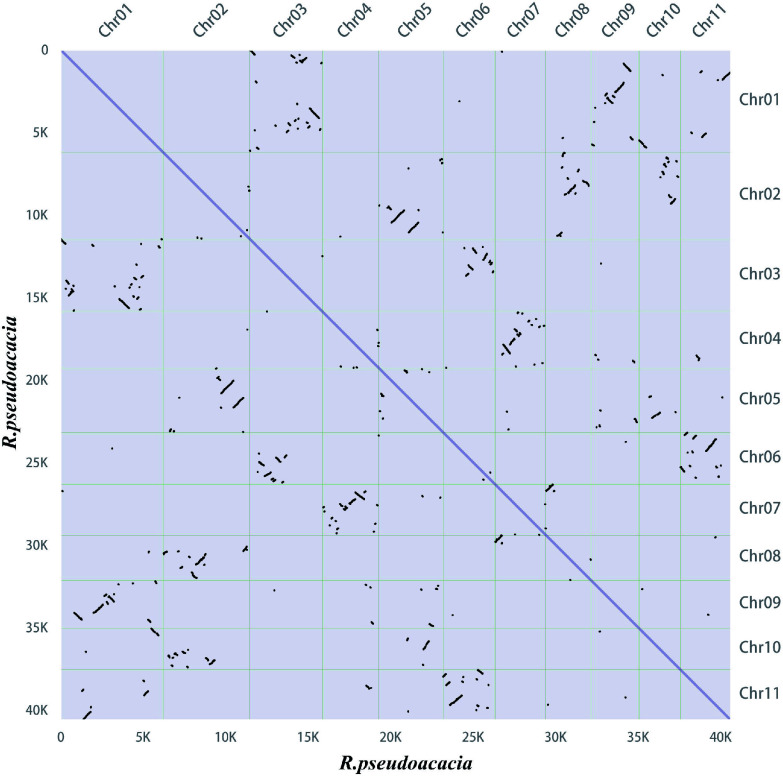
Synteny dot plot within the *Robinia pseudoacacia* genome. In the upper right section, the x-axis and y-axis represent the chromosomes (chr01–11) of *R. pseudoacacia*, arranged in order from left to right and top to bottom according to chromosome numbers. In the lower left section, the x-axis and y-axis indicate lengths ranging from 0–40 K. Each dot represents one homologous block, determined by the positions of genes on the chromosomes.

**Figure 4:**
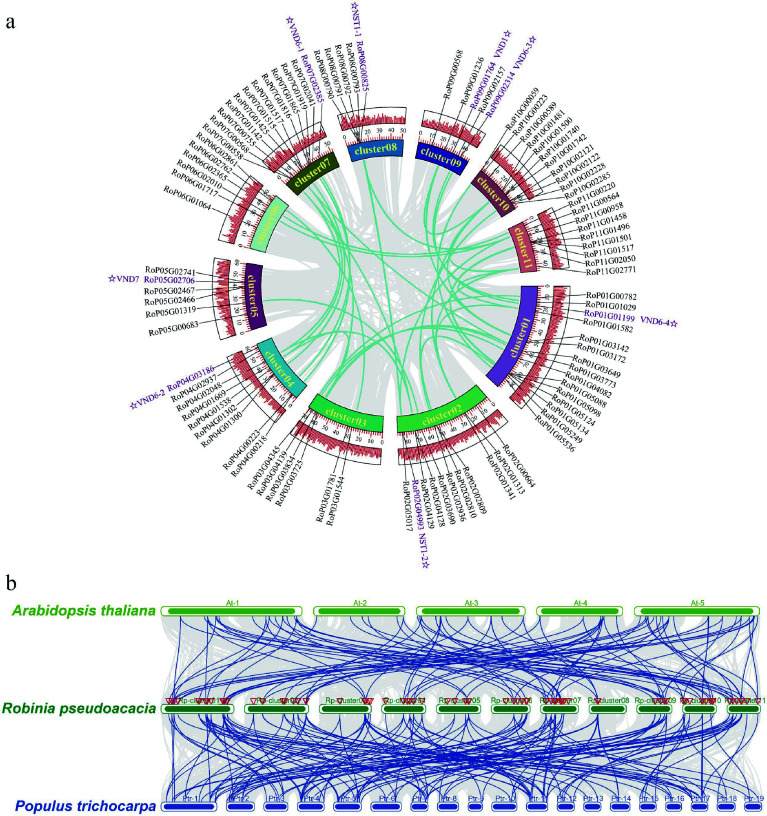
Intra-species and inter-species collinearity analysis of NAC genes in *Robinia pseudoacacia*, *Arabidopsis thaliana*, and *Populus trichocarpa*. (a) Intra-specific collinearity analysis of NAC genes in *Robinia pseudoacacia*. (b) Interspecific collinearity analysis of NAC genes among *R. pseudoacacia*, *P. trichocarpa,* and *A. thaliana*.

Gene family members for monolignol biosynthesis were specifically checked, and 359 gene models were identified, encoding eleven enzyme families, including 14 phenylalanine ammonia-lyase (PAL), three cinnamate 4-hydroxylase (C4H), 59 p-coumarate CoA ligase (4CL), 118 hydroxycinnamoyltransferase (HCT), eight *p*-coumaroyl-CoA 3-hydroxylase (C3H), 21 caffeoyl shikimate esterase (CSE) genes, nine caffeoyl-CoA *O*-methyltransferase (CCoAOMT) genes, 37 cinnamoyl-CoA reductase (CCR), 33 coniferaldehyde 5-hydroxylase (CAld5H), 65 caffeic acid 3-*O*-methyltransferases (COMT), and 22 cinnamyl alcohol dehydrogenase (CAD) genes (Supplementary Data 3). A total of 4,467 syntenic gene pairs were identified through intragenomic synteny analysis of the *R. pseudoacacia* genome (Fig. 3). Sequence alignment revealed that among these 359 genes, 45 pairs were whole-genome duplication (WGD) gene pairs (Supplementary Table S5). NAC TF genes have been identified as master regulators of secondary cell wall biosynthesis in the stems of Arabidopsis and poplar^[45,46]^. In Arabidopsis, VASCULAR-RELATED NAC-DOMAIN (VND) proteins regulate vessel differentiation^[47−49]^, and NAC SECONDARY WALL THICKENING PROMOTING FACTOR (NST) / SECONDARY WALL-ASSOCIATED NAC DOMAIN PROTEIN (SND) proteins regulate fiber differentiation^[50]^. A total of 95 NAC genes were identified in the *R. pseudoacacia* genome. Among these 95 NAC genes, 27 pairs belonged to WGD gene pairs (Supplementary Table S5). Collinearity analysis revealed that the NAC family harbors 32 pairs of segmentally duplicated genes within *R. pseudoacacia* (Fig. 4a). Comparative analyses with Arabidopsis and *P. trichocarpa* showed that *R. pseudoacacia* shares 48 and 62 collinear segments with the two species, respectively, indicating that this gene family exhibits a certain degree of conservation during evolution and that segmental duplication is an important driver for the expansion of the NAC gene family in *R. pseudoacacia* (Fig. 4b).

### Microstructure of stipular spines

**Figure 5:**
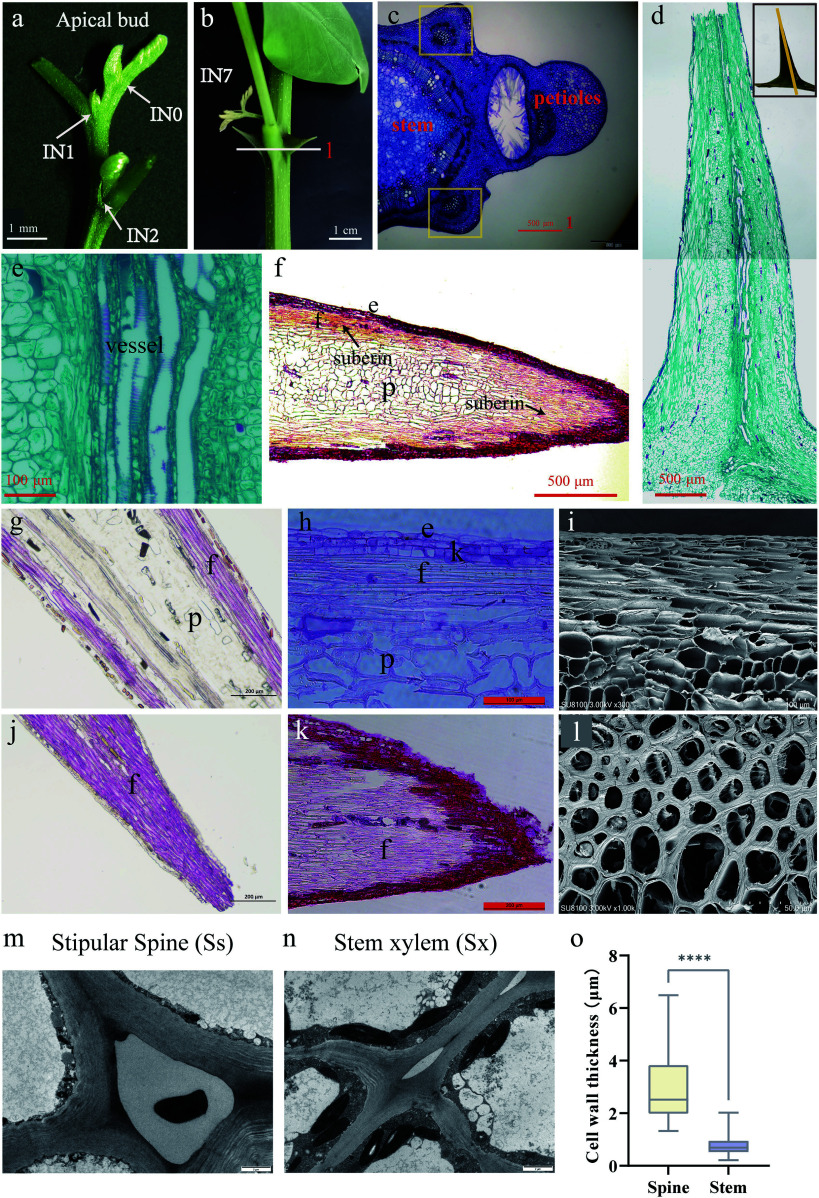
Stipular spines in *Robinia pseudoacacia* variety AGT*.* (a) The apical bud of AGT, a large-spined clonal line of *R. pseudoacacia.* (b) The large stipular spines of AGT(IN7). (c) Cross-section of the internode, scale bar 500 μm. (d) Longitudinal section of stipular spine, scale bar 500 μm. (e) vascular tissue of stipular spine. Scale bar 100 μm. (f) Longitudinal section of perennial stipular spine, scale bar 500 μm. (g) Longitudinal section of the middle part of stipular spines stained with phloroglucinol, scale bar 200 μm. (h) Longitudinal section of the middle part of stipular spines stained with safranin, scale bar 100 μm. (i) Electron micrograph of the longitudinal section of stipular spines, scale bar 100 μm. (j) Longitudinal section of the tip part of stipular spines stained with phloroglucinol, scale bar 200 μm. (k) Longitudinal section of the tip part of stipular spines stained with safranin, scale bar 200 μm. (l) Electron micrograph of the cross-section of stipular spines, scale bar 500 μm. (m) Transmission electron microscope (TEM) images of stipular spine cells. (n) Transmission electron microscope (TEM) images of stem cells. (o) Cell wall thickness of fiber cells in stem xylem (Sx) and stipular spine (Ss). The asterisks indicate the significance (**** *p* < 0.001, the Student’s t-test).

The stipular spines of *R. pseudoacacia* become visible at the second IN2 of the stem, and those on the IN7 of the large-spined variety AGT appeared rigid and measured 2 cm in length (Fig. 5a, b). To investigate the hardness of the stipular spines, anatomical observation was performed on both transverse and longitudinal sections of mature stipular spines at IN7. In the cross sections, each stipular spine contained one vascular bundle, inside which vessel elements exhibited lignified and thickened secondary walls in a spiral manner (Fig. 5c−f). In the longitudinal sections stained with phloroglucinol or safranin, the fiber cells were stained red, which were mainly distributed beneath the epidermal cells in the tip of the stipular spines (Fig. 5g, h, j and k). While in the sections stained with safranin, numerous simple pits were observed on the cell walls of fiber cells, and the fiber cells beneath the epidermis at the stipular spine apex exhibited suberization (Fig. 5h, k). Under SEM, a thickened wall of these fiber cells can be clearly observed (Fig. 5i, l). The measurement under TEM showed that fiber cell walls in stipular spines were significantly thicker than the fiber cells in stem xylem (*p* < 0.0001), with an average wall thickness of 2.931 μm in stipular spines and 0.7807 μm in xylem (Fig. 5m−o).

### Lignin accumulation in stipular spines

**Figure 6:**
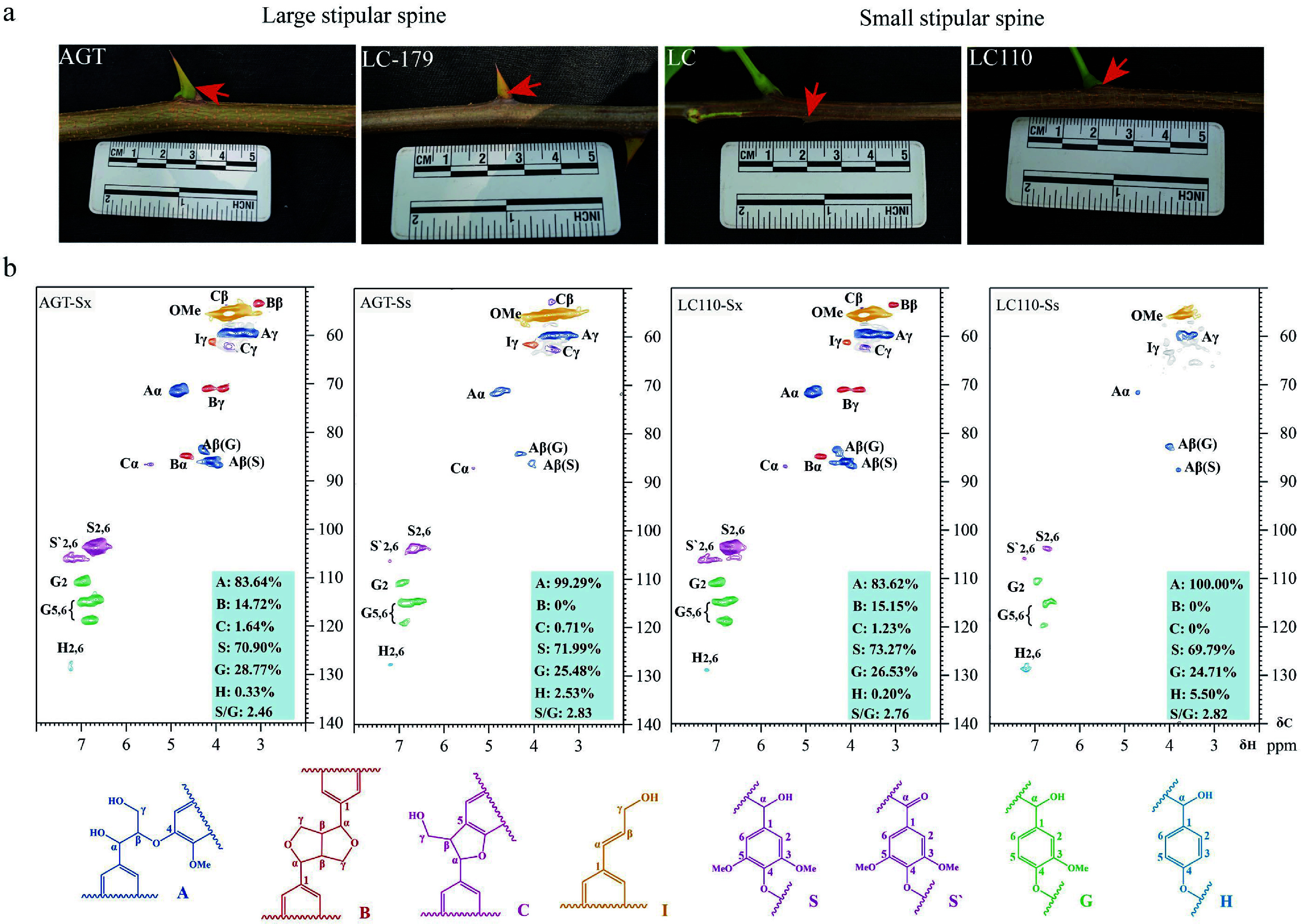
Lignin accumulation in stipular spines and stems of *Robinia pseudoacacia.* (a) Photos show the stipular spines in large-spined (AGT and LC179) and small-spined (LC and LC110) varieties. (b) NMR analysis of stem xylem (Sx) and stipular spine (Ss) in the black locust varieties AGT and LC110.

Lignin is one of the major components of secondary cell walls of vessel and fiber cells^[51,52]^. To investigate the lignin deposition and its contribution to the hardness of stipular spines, lignin content of stipular spines was determined in four varieties (AGT, LC179, LC, and LC110), with large (AGT and LC179) and small (LC and LC110) stipular spines, respectively (Fig. 6a, Table 1). The analysis showed that large and small stipular spines, all had high lignin content, with 48.57%, 63.71%, 53.81%, and 54.01% in AGT, LC, LC110, and LC179, respectively (Table 1). While the lignin content in stem xylem was relatively lower than that in stipular spines, with 27.99%, 24.57%, 26.52%, and 25.09% in these four varieties, respectively (*p* < 0.001).

**Table 1:** The total lignin content of stem xylem and stipular spines in four varieties of *Robinia pseudoacacia* trees. Sx: Stem xylem; Ss: Stipular spine.

Sample	Klason lignin (%)	Acid-soluble lignin (%)	Total lignin (%)	Significance (Sx vs Ss)
AGT-Sx	22.43	5.56	27.99 ± 0.35	a
AGT-Ss	43.36	5.21	48.57 ± 0.55	b
LC-Sx	19.31	5.26	24.57 ± 0.26	a
LC-Ss	58.45	5.26	63.71 ± 0.33	b
LC110-Sx	20.78	5.74	26.52 ± 0.51	a
LC110-Ss	48.42	5.39	53.81 ± 0.44	b
LC179-Sx	19.87	5.22	25.09 ± 0.28	a
LC179-Ss	48.81	5.20	54.01 ± 0.41	b
Statistical analysis was performed using paired *t*-test to compare lignin content between stem xylem (Sx) and stipular spine (Ss) of the same variety. AGT and LC179 are the varieties with large stipular spines, and LC and LC110 are the varieties with small stipular spines. Total lignin contents are presented as mean values ± SD, and the error bars represent standard deviation derived from three independent measurements. Different lowercase letters (a, b) in the 'Significance (Sx vs Ss)' column indicate highly significant differences at the *p* ≤ 0.001 level (consistent with the obvious numerical gap between Sx and Ss in each variety).				

Nuclear magnetic resonance (NMR) was further used to determine lignin composition in both stipular spines and stems of AGT and LC110. Both guaiacyl (G) and syringyl (S) lignin units were detected, with a higher percentage of S-units, and extremely trace amounts of *p*-hydroxyphenyl (H) lignin could be detected in both samples (Fig. 6b). The percentage of S-units in the stipular spines of the four varieties (AGT, LC179, LC, and LC110) was approximately at the same level (69.79%–73.27%) as the stems, representing a S/G ratio of 2.5 (Fig. 6b). The linkage types of lignin directly reflect its structural complexity, with *β*-O-4 linkage being the most abundant in lignin, whereas *β*-*β* and *β*-5 are minor ones. The S/G ratio reflects the relative proportion of syringyl and guaiacyl units in lignin; a higher proportion of S-units generally renders lignin more susceptible to chemical/biological degradation. Compared with stems, the stipular spines of AGT (AGT-Ss), the lignin structure relied more on *β*-O-4 linkages with an increased proportion of S-units; in the stipular spines of LC110 (LC110-Ss), the relative proportion of G-units in the lignin structure increased, and the *β*-*β* and *β*-5 linkages were significantly simplified (Supplementary Table S6).

### Gene expression analysis during stipular spine development

To investigate the molecular basis for lignin biosynthesis in stipular spines, high-throughput RNA sequencing was performed in developing tissues of xylem, phloem, leaves, and stipular spines IN1–4 that represented four developmental stages (Ss1–4). A total of 1,627 DEGs were identified in the stipular spines from IN2–4 of AGT (AGT-Ss2, 3, and 4), compared to AGT-Ss1. While 1,751 DEGs were identified in LC110-Ss2–4 compared to LC110-Ss1. This large number of DEGs indicates high-level transcriptional changes occurring during stipular spine development.

RNA-seq analysis identified 428 common DEGs in AGT and LC110 stipular spine (AGT-Ss and LC110-Ss) compared to BL01 leaves (BL01-Le) (Log_2_FC > 1, q < 0.01) (Supplementary Fig. S6). GO analysis showed that these genes were significantly enriched in a series of basic biological processes, including xylan metabolic process, xylan biosynthetic process, plant-type secondary cell wall biogenesis, cell wall biogenesis, and hemicellulose metabolic process (Supplementary Fig. S7). KEGG analysis identified 20 significantly enriched pathways, including biosynthesis of secondary metabolites, flavonoid biosynthesis, metabolic pathways, and phenylpropanoid biosynthesis etc (Supplementary Fig. S8).

RNA-seq analysis showed that 581 DEGs were shared by AGT-Ss and LC110-Ss (Log_2_FC > 1, q < 0.01) (Supplementary Fig. S6). GO Functional annotation enrichment analysis uncovered that these genes were significantly enriched in five biological processes and one molecular function, including developmental process, cellular carbohydrate biosynthetic process, and carbohydrate biosynthetic process (Supplementary Table S7). KEGG pathways enrichment analysis revealed that these genes were significantly enriched in the biosynthesis of secondary metabolites, valine, leucine and isoleucine degradation, metabolic pathways, and flavonoid biosynthesis (Supplementary Table S7).

RNA-seq analysis revealed that metabolite processes associated with secondary wall biosynthesis occur during stipular development. The expressions of the 359 predicted genes encoding monolignol biosynthetic enzymes were examined in different tissues. Among them, 115 were highly expressed in stipular spines, and 62 were highly expressed in developing xylem (Log_2_FC > 1, q < 0.01), including *RopCCR2a*, *C4H1*, and *C4H2*. Some catalytic steps in the monolignol biosynthetic pathway apparently recruited the same enzymes between xylem and stipular spine, such as RopPAL4a, Rop4CL5, RopCAD1, RopC3H3, RopCAld5H1, and RopHCT1. Some steps involved different enzyme family members between developing xylem and stipular spines. For example, *RopHCT6a*/*35*/*41*, *RopHCT40*, *RopCCoAOMT1b*/*3*, *RopCSE1*, and *RopCCR5a*/*31* were specifically expressed in developing xylem, while *RopCAld5H2*, *RopCOMT1a*/*36*/*52*, *RopCCR18*, *RopC4H3*, *Rop4CL3a*, *RopHCT1*, *Rop4CL5*, and *RopCAD1* were specifically expressed in stipular spines. Generally, more genes were expressed in stipular spines than in xylem, which could explain the higher lignin content in stipular spines than in xylem.

**Figure 7:**
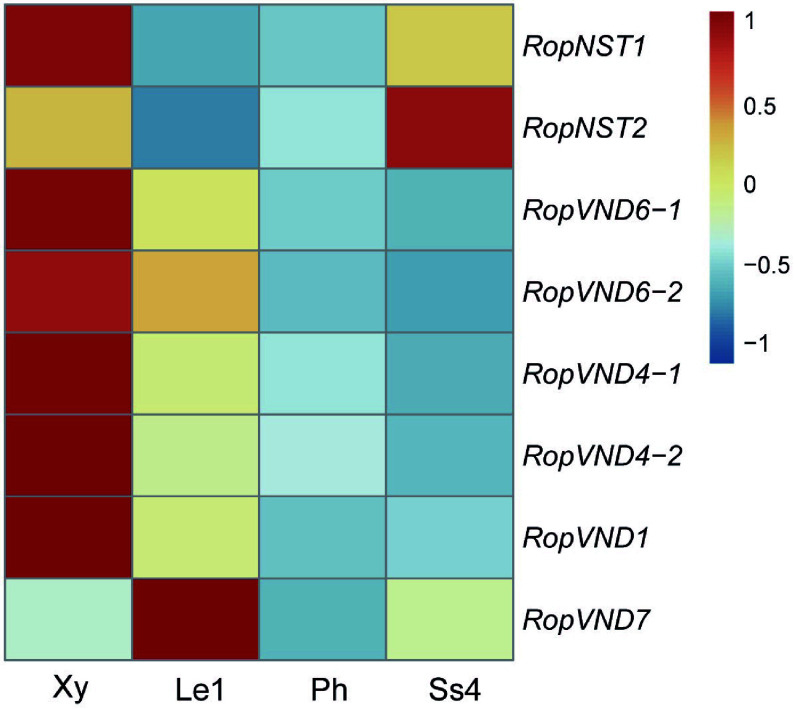
Heat map shows the relative expression levels of six *VND* and two *NST* genes in developing xylem (Xy), leaf stage 1 (Le1), phloem (Ph) and stipular spine stage 4 (Ss4) of *R. pseudoacacia*. Fragments per kilobase million (FPKM) values were normalized using the Z-score method. 'Rop' is the abbreviation of *Robinia pseudoacacia*, representing the species-specific genes of this plant.

The phylogenetic tree constructed using NAC protein sequences from different species (Supplementary Fig. S9) showed that two genes were NST orthologs and six genes were VND orthologs. These two NST genes (*RopNST1* and *NST2*) were significantly upregulated in the stipular spines compared to the leaf. *RopNST1* and four *VND* genes (*RopVND1*, *VND4-1*, *VND4-2*, and *VND6-1*) were differentially expressed in developing xylem compared to phloem and leaf (Fig. 7, Supplementary Data 4), indicating that these five genes are probably the key regulators of secondary wall thickening during vessel and fiber formation in the stem xylem cells of *R. pseudoacacia*. Furthermore, the expression of *RopVND7* was significantly higher in leaves and stipular spines compared to xylem (Fig. 7, Supplementary Data 4).

**Figure 8:**
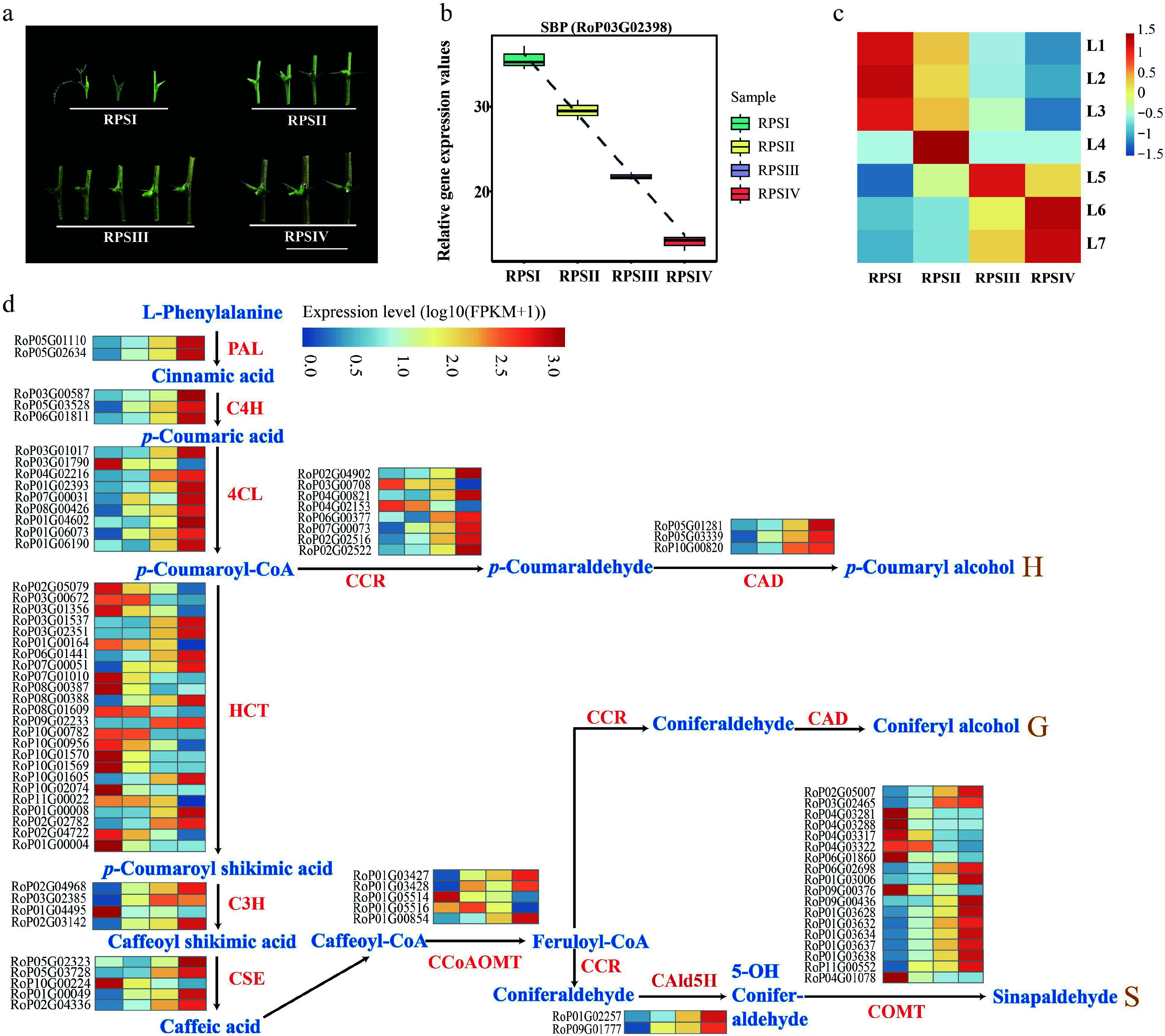
Gene expression analysis in different developmental stage of stipular spines. (a) Four different developmental stages of stipular spines, including RPSI (1^st^ to 3^rd^ pair of stipular spines, counting from top), RPSII (the 5^th^ pair), RPSIII (the 7^th^ pair), and RPSIV (the 10^th^ pair). (b) Relative expression values of *SBP* (RoP03G02398) which was used as the bait gene. (c) The heatmap of normalized TPMs (z-scores color-coded) at each time point is shown. (d) Heat map show the expression levels of differentially expressed enzyme genes of monolignol biosynthetic pathway. Blue color indicates low expression levels and red color indicates high expression levels. Abbreviations used in the figure: PAL, Phenylalanine Ammonia-Lyase; C4H, Cinnamate 4-Hydroxylase; 4CL, 4-Coumarate: CoA Ligase; CCR, Cinnamoyl CoA Reductase; CAD, Cinnamyl Alcohol Dehydrogenase; HCT, Hydroxycinnamoyl-CoA Shikimate/Quinate Hydroxycinnamoyl Transferase; C3H, *p*-Coumarate 3-Hydroxylase; CSE, Coumarate 5-Hydroxylase; CCoAOMT, Caffeoyl CoA *O*-Methyltransferase; CAld5H, Coumaraldehyde 5-Hydroxylase; COMT, Caffeic Acid *O*-Methyltransferase.

**Figure 9:**
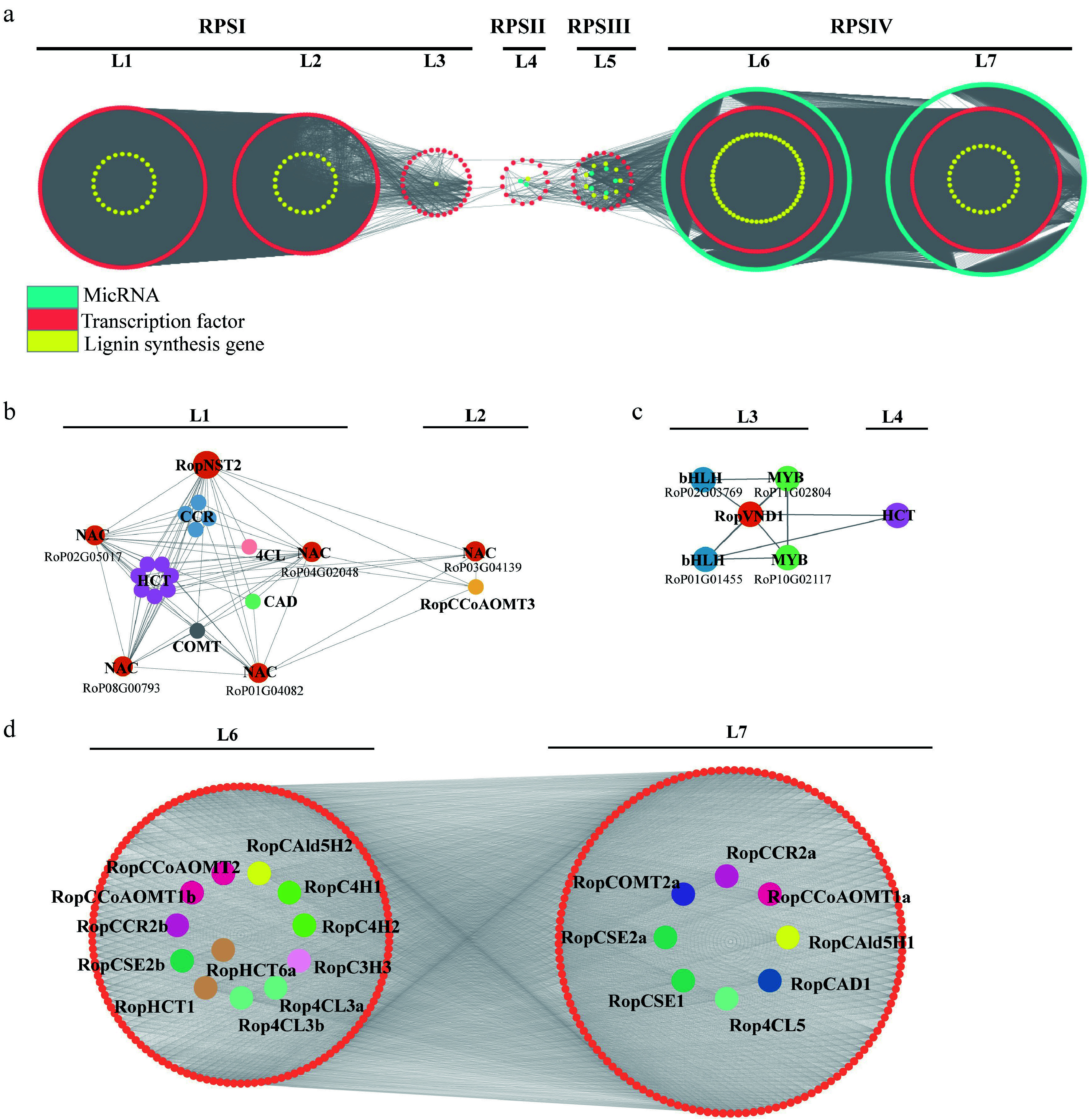
Regulatory subnetwork of lignin biosynthesis. (a) Stipular spine development-related temporal gene co-expression network. L1–L7 represent seven levels based on the expression level of the seed gene. L1–L3 corresponds to the RPSI period, L4 corresponds to the RPSII period, Level 5 corresponds to the RPSIII period, and L6 and L7 corresponds to the RPSIV period. Orange circle indicates the genes related to lignin biosynthesis, yellow circle indicates transcription factors, and turquoise circle indicates miRNA. (b) Subnetwork of lignin biosynthesis-related genes regulated by RopNST2 at L1 and L2. The members for the 4CL, HCT, CCR, CAD, and COMT include *Rop4CL12b*, *RopHCT13b*/*79*/*48*/*14b*/*20*/*86*/*9a*, *RopCCR3b*/*16*/*24*/*5a*, *RopCAD19*, and *RopCOMT37*, respectively. (c) Subnetwork of lignin biosynthesis-related enzyme gene and transcription factors regulated by RopVND1 at L3 and L4. (d) Subnetwork of lignin biosynthesis-related genes at L6 and L7 levels, where orange circles represent transcription factors, and different lignin genes are represented by circles with different colors. The prefix 'Rop' in the gene names is the abbreviation of *Robinia pseudoacacia*.

Whole-transcriptome sequencing was further performed using stipular spines from different internodes of the current-year branch of the AGT variety. These stipular spines were divided into four groups, representing four different developmental stages: RPSI (1^st^ to 3^rd^ pair of stipular spines, counting from top), RPSII (the 5^th^ pair), RPSIII (the 7^th^ pair), and RPSIV (the 10^th^ pair) (Fig. 8a). A total of 2,048 miRNAs, 40,077 genes, and 1,447 TFs were obtained. A TO-GCN was constructed using a SBP family TF (RoP03G02398), which had an expression peak at RPSI and was downregulated over treatment time, as the bait gene, along with 737 TFs, 537 miRNAs, and 158 lignin synthesis-related genes (Figs 8b, 9a). This network consisted of 116,189 edges and 1,898 nodes, and was divided into seven levels corresponding to the developmental timelines of the stipular spines. The assigned levels accurately matched the time-order of DEG expression level changes across the four experimental time points (RPSI, RPSII, RPSIII, and RPSIV) (Fig. 8c), as indicated by the gene expression peaking (i.e., the red boxes along the diagonal). Coupling the peaks of gene expression overlaps between consecutive levels (L1–7) and the time-series samples (RPSI, RPSII, RPSIII, and RPSIV) forms the basis for inferring upstream and downstream gene/miRNA regulatory relationships based on the respective hierarchical network levels. Namely, genes at the upper level might be regulators of genes co-expressed at the same or the next levels, and the latter might regulate related co-expressed genes at the same or lower level, such as the development process, thus forming potential regulatory pathways with a clear hierarchical structure. Analysis of the seven network levels revealed that miRNAs were first upregulated at L4 (corresponding to RPSII) and their abundance was dramatically increased at L6–7 (RPSIV), indicating that miRNAs possibly regulate the later stages of stipular spine formation. Meanwhile, *RopCCoAOMT3*, *RopCOMT2b*, and *RopHCT6b* were expressed at L2. *RopPAL2a/3/4a*, *Rop4CL3a/3b*, *RopCCR2b*, *RopCCoAOMT1b/2*, *RopCSE2b*, *RopHCT1/6a*, *RopCAld5H2*, *RopC3H3*, *RopC4H1*, and *RopC4H2* were expressed at L6 (Fig. 9d). *Rop4CL5*, *RopCAD1*, *RopCCR2a*, *RopCCoAOMT1a*, *RopCOMT2a*, *RopCSE1/2a*, and *RopCAld5H1* were expressed at L7 (Fig. 9d). *RopNST2* was expressed at L1, while *RopVND1* was primarily expressed at L3. *RopNST2* expressed at L1 regulates the expression of *RopCCoAOMT3* at L2, thereby promoting lignin polymerization and cell wall maturation, which is directly linked to the spine hardening phenotype (Fig. 9b, c). In order to investigate the expression pattern of lignin synthesis-related genes during stipular spine development, more than threefold changes of lignin synthesis-related DEGs were screened (Fig. 8d). A total of 83 lignin synthesis pathway genes were identified, including two *PAL*s, three *C4Hs*, nine *4CL*s, eight *CCR*s, three *CAD*s, 24 *HCT*s, four *C3H*s, five *CSE*s, five *CCoAOMT*s, two *CALd5H*s, and 18 *COMT*s.

## Discussion

In this study, a chromosome-level reference genome of *R. pseudoacacia* was assembled with high quality (contig N50 of 1.1 Mb and BUSCO completeness of 97.1%), providing a reliable foundation for dissecting the molecular mechanisms underlying stipular spine morphogenesis. Genome annotation revealed that repetitive sequences account for 52.93% of the genome, with retrotransposons being the dominant component. These may play a role in regulating gene expression during the species' adaptive evolution^[53,54]^. Notably, a total of 359 genes belonging to 11 enzyme families involved in the lignin biosynthetic pathway were identified, far exceeding the numbers reported in Arabidopsis and other plants (Supplementary Data 5). Among these, 45 pairs of genes are derived from WGD, suggesting that WGD may influence lignin biosynthesis capacity. This finding is consistent with phenotypic data showing that lignin content in stipular spines of *R. pseudoacacia* is significantly higher than that in stem xylem (e.g., 48.57% in stipular spines vs 27.99% in stem xylem of AGT), indicating that the WGD events of lignin biosynthesis-related gene families have been involved in the evolutionary process of stipular spine hardening in *R. pseudoacacia*.

Through microstructural observations, spirally lignified vessel elements were observed in the vascular bundle and thick walls in the fiber cells beneath the epidermis. Phloroglucinol staining indicates that lignin is accumulated in these fiber cells, which may contribute to the hardness of the stipular spines. In other studies, suberization in the cell walls of green fibers has been well documented in cotton and russeted apple peel^[55,56]^. It is hypothesized that the synergistic accumulation of suberin and lignin may further enhance the mechanical strength and stress resistance of stipular spines. Analysis of lignin monomer composition showed that the proportion of syringyl (S)-type lignin in stipular spines was as high as 69.79%–73.27% (with an S/G ratio of approximately 2.5). This result is closely associated with the spine-specific high expression of lignin biosynthesis-related genes in transcriptomic data: among the 115 lignin biosynthesis-related genes that were highly expressed in stipular spines, the specific expression of *RopCAld5H2* and *RopCOMT1a* may be the key drivers of the high accumulation of S-type lignin. The COMT gene encodes a caffeic acid *O*-methyltransferase, a core enzyme for S-type lignin synthesis, whose activity directly determines the S/G ratio^[57]^. During the domestication of *Fagopyrum tataricum*, selection pressure on the *COMT* gene led to an increased proportion of S-type lignin and a decrease in pericarp hardness in cultivated varieties^[58]^. In contrast, the high expression of *COMT* homologs in stipular spines may represent an opposing evolutionary strategy, optimizing spine toughness by increasing the proportion of S-type lignin. This regulatory pattern further reflects the flexibility of the lignin biosynthetic pathway in the functional specialization of different plant organs.

At the transcriptional regulation level, NAC family TFs play a particularly prominent role. Members of the NAC TF family, including *RopNST1/2* and *RopVND1/4/6*, are differentially expressed in stipular spines, showing functional conservation with their homologous genes that regulate secondary wall synthesis in Arabidopsi*s*^[59−62]^. Expression analysis indicated that *RopNST1/2* were significantly upregulated in stipular spines, while *RopVND1/4/6* were differentially expressed in stem xylem, suggesting that these genes regulate secondary wall thickening in stipular spine fibers and stem xylem vessels, respectively. Notably, this regulatory pattern is highly conserved with the function of NAC genes in other plants: in Arabidopsis, AtNST1/2 directly activate lignin biosynthesis genes, while AtVND1/4/6 specifically regulate secondary wall thickening in vessel elements^[59,61]^. Similarly, Ren et al. identified a comparable hierarchical regulatory network in citrus stem thorns—where the MYB TF SST1 establishes a 'MYB→NAC→lignin synthase' pathway by directly binding to the promoters of *NST1* and *SND1* and activating their expression, ultimately driving the accumulation of G/S-type lignin and secondary wall thickening at the thorn tips^[10]^. In this study, the high expression level and functional prediction of *RopNST1/2* in stipular spines are fully consistent with the role of *NST1/SND1* in citrus, indicating that NAC genes *NST*/*SND* may be universal core switches for lignification in plant thorn organs, and the activation mechanism of their downstream targets lignin biosynthesis genes (such as *PAL*, *CCR*, and *CAD*) exhibits cross-species conservation between stem thorns (citrus) and stipular spines (*R. pseudoacacia*), further verifying the evolutionary stability of the regulatory network for plant secondary wall synthesis. In this study, the development of *R. pseudoacacia* stipular spines also relies on the precise regulation of organ identity (e.g., the differentiation of stipule primordia into spines). It is hypothesized that the expression of stipular spine-specific lignification genes may also be regulated by TCP family genes. In future studies, this conserved regulatory pattern of 'organ identity genes→lignification regulators' can be verified by screening *R. pseudoacacia* TCP genes and detecting their binding to the promoters of *RopNST1/2* or lignin biosynthesis-related genes, thereby improving the molecular network of stipular spine development.

In summary, the hardening of thorn organs and enhancement of their defensive functions are achieved through the conserved 'NAC core factors→lignin biosynthesis genes' module. This characteristic of 'different origins with similar regulatory mechanisms' represents a classic case of convergent evolution in plant thorn organs, providing a new molecular perspective for understanding the evolutionary diversity of plant defensive organs.

TO-GCN analysis showed that miRNAs are significantly enriched in the late stages of spine development (L6 and L7), potentially regulating stipular spine maturation by targeting lignin biosynthetic enzyme genes. This finding is analogous to the mechanism by which the miR172a-SNB-MYB30 module regulates lignin deposition in rice: miRNAs relieve the repression of *MYB30* by inhibiting the TF SNB, thereby activating the lignin synthesis pathway^[63]^. Similarly, in *R. pseudoacacia*, miRNAs may regulate the proportion of lignin monomers or polymer structure in the late developmental stages, for instance by targeting the expression of *COMT* or *CAD*, thereby optimizing the mechanical properties of spines.

These findings link the structural specialization of stipular spines with the temporal dynamics of gene expression, providing an integrated structure-molecule-regulation model for understanding the morphogenesis of plant defensive organs.

## Conclusions

In this study, the morphogenesis and lignification of stipular spines in *R. pseudoacacia* were investigated by integrating chromosome-level genome assembly, cytological observation, lignin quantification, and transcriptomic analysis. A high-quality reference genome of *R. pseudoacacia* (681.6 Mb in size, with a Contig N50 of 1.1 Mb) was constructed, and 359 lignin biosynthesis-related genes were identified (including 14 PAL, three C4H, and 22 CAD genes). Transcriptomic and TO-GCN analyses showed that the NAC TF genes *RopNST1/2* were significantly upregulated in stipular spines, which could directly activate the expression of lignin biosynthesis. The genome and key genes identified in this study directly provide targets for the genetic improvement of defensive traits in forest trees.

## SUPPLEMENTARY DATA

Supplementary data to this article can be found online.

## Data Availability

Whole-genome sequencing and Hi-C sequencing data were deposited to the China National Center for Bioinformation under Bioproject PRJCA046164. RNA-seq and whole-transcriptome data were deposited to the China National Center for Bioinformation Genome Sequence Archive under accession number PRJCA046469. The genome assembly and annotation files are available from the Figshare database (doi: 10.6084/m9.figshare.30594863)
