# Supplementary material for: Genome and transcriptomics provide insights on stipular spine morphogenesis in Robinia pseudoacacia
Source: For Res (Fayettev). 2026 Jan 31;6:e003. doi: 10.48130/forres-0026-0003 (PMC13187913; doi:10.48130/forres-0026-0003)
Supplement: Supplementary file 1 — Supplementary data to this article can be found online. [file forres-6-1-e003-Supplementary.zip › 10.48130_forres-0026-0003-Suppl-FigureS7.pdf]

**Table S7.** GO and KEGG enrichment analysis of differentially expressed genes in spines and leaves together. BP: biological process. Q-value less than 0.01 (for GO) and 0.05 (for KEGG) represented significant enrichment.

**GO enrichment**

| Category | GO ID | Description | P-value | Q-value | Ratio |
|---|---|---|---|---|---|
| BP | GO:0032502 | developmental process | 4.85E-11 | 1.16E-08 | 0.081 |
| BP | GO:0006355 | regulation of transcription, DNA-templated | 0.000136 | 0.003324 | 0.015 |
| BP | GO:0051252 | regulation of RNA metabolic process | 0.000136 | 0.003324 | 0.015 |
| BP | GO:1903506 | regulation of nucleic acid-templated transcription | 0.000136 | 0.003324 | 0.015 |
| BP | GO:2001141 | regulation of RNA biosynthetic process | 0.000136 | 0.003324 | 0.015 |
| BP | GO:0019219 | regulation of nucleobase-containing compound metabolic process | 0.000145 | 0.003324 | 0.015 |
| BP | GO:0009889 | regulation of biosynthetic process | 0.000147 | 0.003324 | 0.015 |
| BP | GO:0010556 | regulation of macromolecule biosynthetic process | 0.000147 | 0.003324 | 0.015 |
| BP | GO:0031326 | regulation of cellular biosynthetic process | 0.000147 | 0.003324 | 0.015 |
| BP | GO:2000112 | regulation of cellular macromolecule biosynthetic process | 0.000147 | 0.003324 | 0.015 |
| BP | GO:0051171 | regulation of nitrogen compound metabolic process | 0.000153 | 0.003324 | 0.015 |
| BP | GO:0010468 | regulation of gene expression | 0.000168 | 0.003346 | 0.015 |
| BP | GO:0031323 | regulation of cellular metabolic process | 0.00023 | 0.004089 | 0.014 |
| BP | GO:0080090 | regulation of primary metabolic process | 0.00024 | 0.004089 | 0.014 |
| BP | GO:0060255 | regulation of macromolecule metabolic process | 0.000272 | 0.004337 | 0.014 |
| BP | GO:0019222 | regulation of metabolic process | 0.000381 | 0.005684 | 0.014 |
| BP | GO:0006351 | transcription, DNA-templated | 0.000579 | 0.007416 | 0.013 |
| BP | GO:0097659 | nucleic acid-templated transcription | 0.000579 | 0.007416 | 0.013 |
| BP | GO:0032774 | RNA biosynthetic process | 0.00059 | 0.007416 | 0.013 |
| BP | GO:0007275 | multicellular organism development | 0.00074 | 0.008838 | 0.041 |
| BP | GO:0044707 | single-multicellular organism process | 0.000796 | 0.009054 | 0.040 |

**KEGG enrichment**

| Pathway | Ko ID | P-value | Q-value | Ratio |
|---|---|---|---|---|
| Valine, leucine and isoleucine degradation | ko00280 | 0.000247 | 0.011111 | 0.056 |
| Fatty acid elongation | ko00062 | 0.001133 | 0.024237 | 0.062 |
| Limonene and pinene degradation | ko00903 | 0.001616 | 0.024237 | 0.143 |
| Biosynthesis of secondary metabolites | ko01110 | 0.002483 | 0.027938 | 0.008 |
