# Supplementary material for: Genome and transcriptomics provide insights on stipular spine morphogenesis in Robinia pseudoacacia
Source: For Res (Fayettev). 2026 Jan 31;6:e003. doi: 10.48130/forres-0026-0003 (PMC13187913; doi:10.48130/forres-0026-0003)
Supplement: Supplementary file 1 — Supplementary data to this article can be found online. [file forres-6-1-e003-Supplementary.zip › 10.48130_forres-0026-0003-Suppl-FigureS8.pdf]

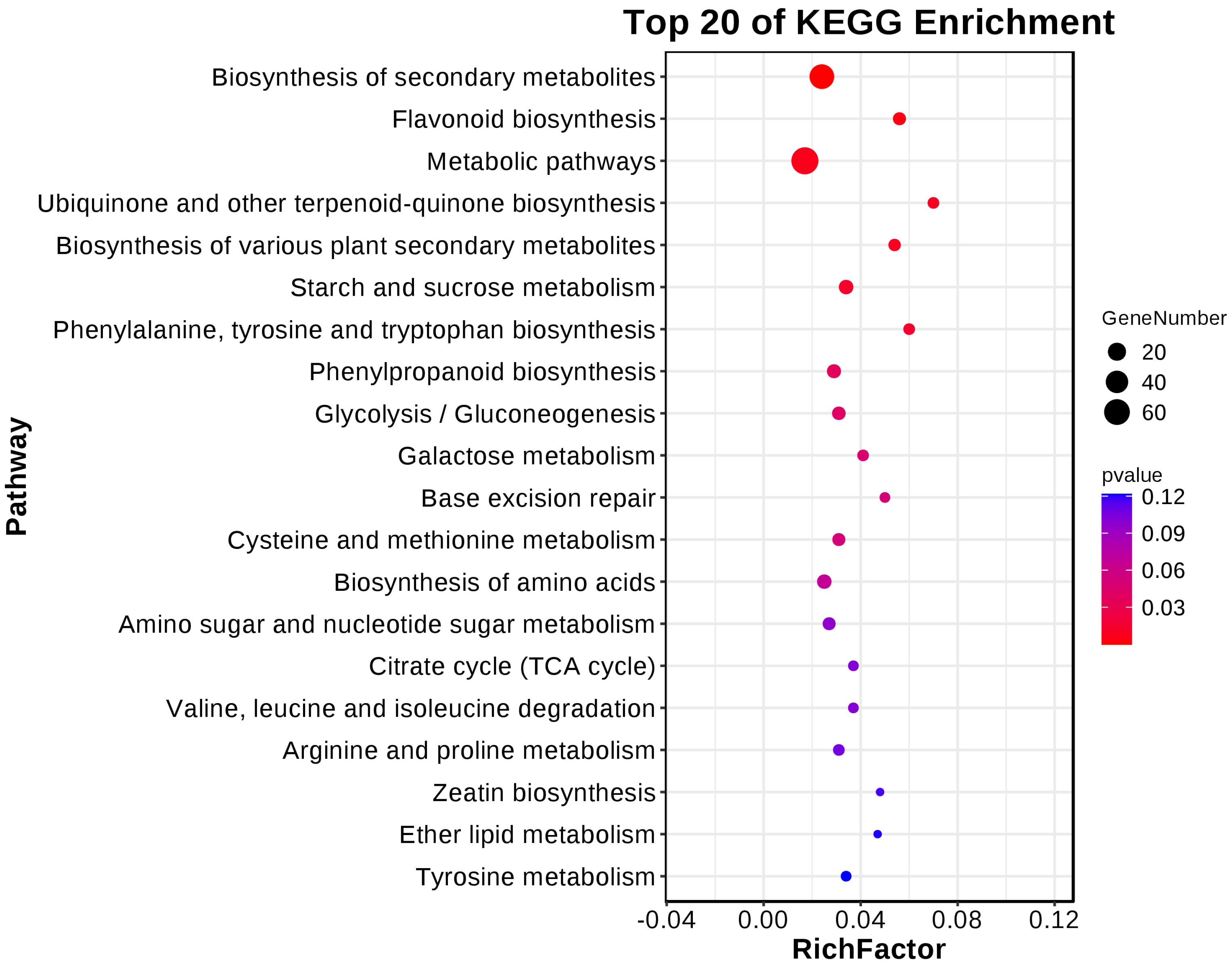

Fig.S8 The top 20 pathways of KEGG enrichment were identified for the 428 differentially expressed genes in stipule spine (AGT-Ss, LC110-Ss) compared to leaf (BL01-Le). Ss: stipule spine, Le: leaf.
