# Supplementary material for: Genome and transcriptomics provide insights on stipular spine morphogenesis in Robinia pseudoacacia
Source: For Res (Fayettev). 2026 Jan 31;6:e003. doi: 10.48130/forres-0026-0003 (PMC13187913; doi:10.48130/forres-0026-0003)
Supplement: Supplementary file 1 — Supplementary data to this article can be found online. [file forres-6-1-e003-Supplementary.zip › 10.48130_forres-0026-0003-Suppl-FigureS9.pdf]

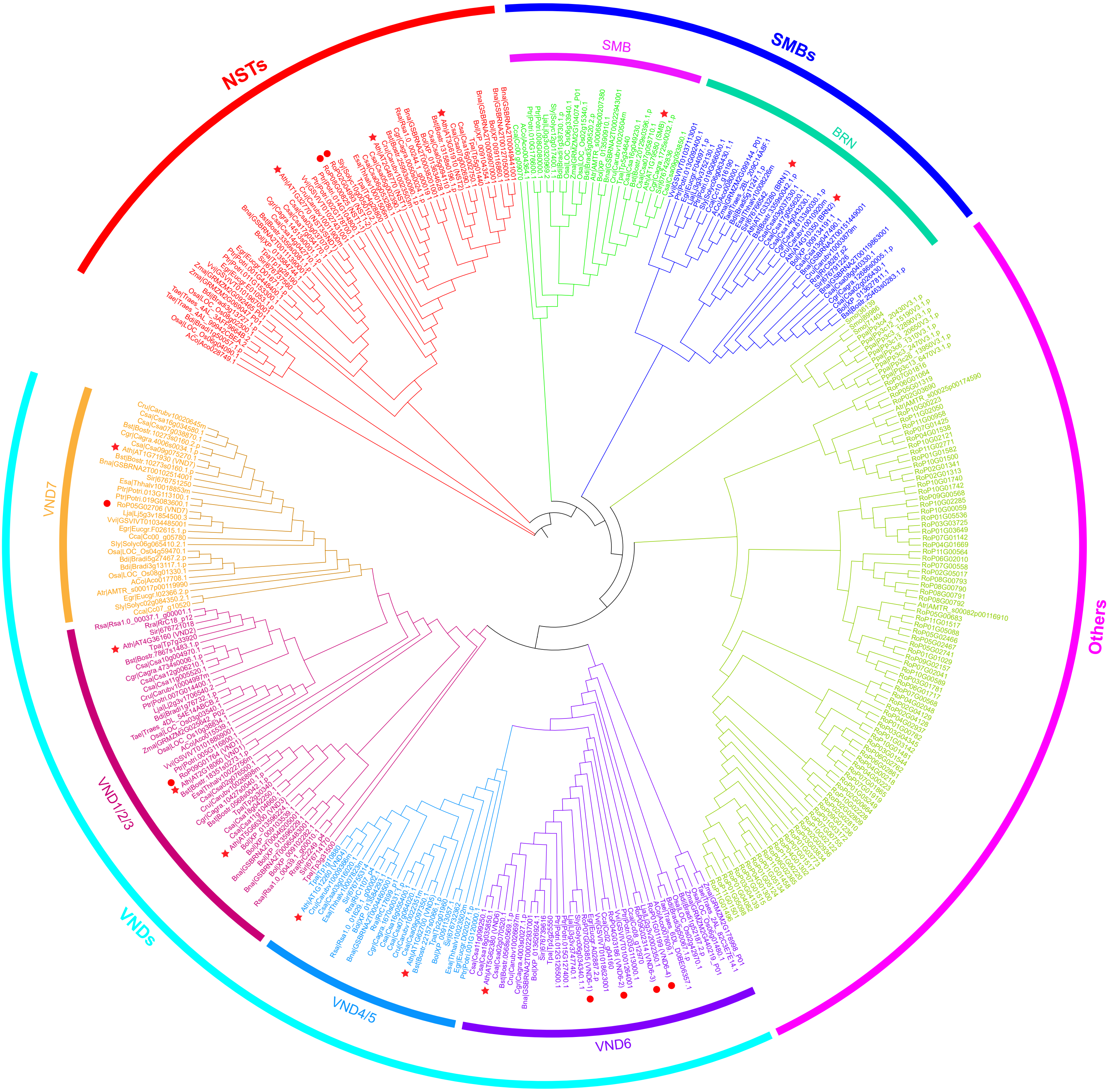

Fig. S9 Phylogenetic analysis of NAC proteins in different plant species. NAC protein sequences are from *Arabidopsis thaliana* (Ath), *Amborella trichopoda* (Atr), *Ananas comosus* (Ao), *Oryza sativa* (Osa), *Populus trichocarpa* (Ptr), *Vitis vinifera* (Vvi), *Zea mays* (Zma), *Triticum aestivum* (Tae), *Eucalyptus grandis* (Egr), *Coffea canephora* (Cca), *Solanum lycopersicum* (Sly), *Lotus japonicas* (Lja), *Brachypodium distachyon* (Bdi), *Physcomitrium patens* (Ppa), *Selaginella moellendorffii* (Smo), *Boechera stricta* (Bst), *Brassica napus* (Bna), *B.rassica oleracea* (Bol), *Camelina sativa* (Csa), *Capsella grandiflora* (Cgr), *C.apsella rubella* (Cru), *Eutrema salsugineum* (Esa), *Raphanus raphanistrum* (Rra), *R.aphanus sativus* (Rsa), *Sisymbrium irio* (Sir), and *Thellungiella parvula* (Tpa). Protein sequences were aligned by MUSCLE and the phylogenetic tree was constructed using RAxML using a maximum likelihood method with 300 bootstrap replicates. The genes marked with red stars and circles were *Arabidopsis thaliana* and *R. pseudoacacia* genes.
