# Supplementary material for: Genome and transcriptomics provide insights on stipular spine morphogenesis in Robinia pseudoacacia
Source: For Res (Fayettev). 2026 Jan 31;6:e003. doi: 10.48130/forres-0026-0003 (PMC13187913; doi:10.48130/forres-0026-0003)
Supplement: Supplementary file 1 — Supplementary data to this article can be found online. [file forres-6-1-e003-Supplementary.zip › 10.48130_forres-0026-0003-Suppl-TableS1.pdf]

Table S1. Statistics of contigs and scaffolds level assembly

**Items**	**Nanopore Assembly**	**Hi-C Assembly**
Assembly size (Mbp)	678.9	681.6
No. of contigs (scaffolds)	1210	1267
Maximum length (bp)	8,368,201	8,368,201
N90 (bp)	244,306	238,264
N50 (bp)	1,092,034	1,092,390
