# Supplementary material for: Genome and transcriptomics provide insights on stipular spine morphogenesis in Robinia pseudoacacia
Source: For Res (Fayettev). 2026 Jan 31;6:e003. doi: 10.48130/forres-0026-0003 (PMC13187913; doi:10.48130/forres-0026-0003)
Supplement: Supplementary file 1 — Supplementary data to this article can be found online. [file forres-6-1-e003-Supplementary.zip › 10.48130_forres-0026-0003-Suppl-TableS2.pdf]

Table S2. Statistics of chromosome length

	Contigs.N	Length(bp)
Chr01	142	99,653,780
Chr02	160	88,099,579
Chr03	110	71,279,49
Chr04	142	60,641,388
Chr05	82	61,024,038
Chr06	105	53,316,467
Chr07	106	52,004,788
Chr08	109	51,387,962
Chr09	98	48.569,821
Chr10	101	46,699,428
Chr11	55	46,306,209
Total	1210	678,982,923
