# Supplementary material for: Genome and transcriptomics provide insights on stipular spine morphogenesis in Robinia pseudoacacia
Source: For Res (Fayettev). 2026 Jan 31;6:e003. doi: 10.48130/forres-0026-0003 (PMC13187913; doi:10.48130/forres-0026-0003)
Supplement: Supplementary file 1 — Supplementary data to this article can be found online. [file forres-6-1-e003-Supplementary.zip › 10.48130_forres-0026-0003-Suppl-TableS3.pdf]

Table S3. The completeness of genome assembly and gene annotation using BUSCO pipeline

**Description**	**Number**	**Percentage (%)**
	Genome	Protein-coding genes
Complete BUSCOs (C)	1567(97.1)	1267(92.2)
Complete and single-copy BUSCOs (S)	1453(90.0)	1057(76.9)
Complete and duplicated BUSCOs (D)	114(7.1)	210(15.3)
Fragmented BUSCOs (F)	21(1.3)	69(5.0)
Missing BUSCOs (M)	26(1.6)	39(2.8)
Total BUSCO groups searched	1375(100)	1375(100)
