# Supplementary material for: Genome and transcriptomics provide insights on stipular spine morphogenesis in Robinia pseudoacacia
Source: For Res (Fayettev). 2026 Jan 31;6:e003. doi: 10.48130/forres-0026-0003 (PMC13187913; doi:10.48130/forres-0026-0003)
Supplement: Supplementary file 1 — Supplementary data to this article can be found online. [file forres-6-1-e003-Supplementary.zip › 10.48130_forres-0026-0003-Suppl-TableS4.pdf]

Table S4. Statistics of repeat sequence in *Robinia pseudoacacia*

**classifications**	**Length (bp)**	**% of repeats**	**% of genome**
Total repeat fraction	359,370,747	100.00	52.93
Class I: Retroelement	323,950,000	90.14	47.71
LTR Retrotransposon	219,570,253	61.10	32.34
Ty1/Copia	67,414,894	18.76	9.93
Ty3/Gypsy	97,472,289	27.12	14.36
Other	82,293,669	22.90	12.12
non-LTR Retrotransposon	16,374,947	4.56	2.41
LINE	16,099,840	4.48	2.37
SINE	275,107	0.08	0.04
unclassified retroelement	88,004,800	24.49	12.96
Class II: DNA Transposon	35,420,747	9.86	5.22
CMC	8,592,573	2.39	1.27
hAT	15,676,956	4.36	2.31
Sola-1	858	0.00	0.00
TcMar	155,683	0.04	0.02
PIF/Harbinger	5,604,174	1.56	0.83
Other	5,390,503	1.50	0.79
