# Supplementary material for: Genome and transcriptomics provide insights on stipular spine morphogenesis in Robinia pseudoacacia
Source: For Res (Fayettev). 2026 Jan 31;6:e003. doi: 10.48130/forres-0026-0003 (PMC13187913; doi:10.48130/forres-0026-0003)
Supplement: Supplementary file 1 — Supplementary data to this article can be found online. [file forres-6-1-e003-Supplementary.zip › 10.48130_forres-0026-0003-Suppl-TableS5.pdf]

Table S5. 72 pairs of WGD genes related to lignin biosynthetic pathway in *R. pseudoacacia* genome.

Gene_1	Gene_2	Gene family	Gene_1	Gene_2	Gene family
RoP01G00853	RoP01G03427	CCoAOMT	RoP02G05009	RoP11G00552	COMT
RoP06G02336	RoP11G00206	4CL	RoP03G02041	RoP03G02096	HCT
RoP01G00389	RoP03G02588	4CL	RoP04G02153	RoP07G00642	CCR
RoP01G04495	RoP03G02385	C3H	RoP03G02111	RoP06G01508	HCT
RoP01G04551	RoP03G02431	4CL	RoP03G01537	RoP06G02879	HCT
RoP01G00771	RoP03G02834	CSE	RoP04G02215	RoP05G01079	4CL
RoP01G05422	RoP09G02505	4CL	RoP04G01109	RoP07G01288	CCR
RoP01G02793	RoP09G01317	CAD	RoP04G03009	RoP09G02612	4CL
RoP01G05513	RoP10G00042	CCoAOMT	RoP04G02601	RoP09G00272	4CL
RoP02G04712	RoP02G05081	HCT	RoP02G04716	RoP02G05079	HCT
RoP02G00597	RoP05G03500	CSE	RoP05G01080	RoP07G00690	4CL
RoP02G03749	RoP05G01281	CAD	RoP05G02634	RoP07G00711	PAL
RoP02G03920	RoP05G01110	PAL	RoP05G02900	RoP10G00956	HCT
RoP02G04336	RoP05G02323	CSE	RoP05G02925	RoP10G00904	4CL
RoP02G04722	RoP05G01922	HCT	RoP02G05009	RoP11G00552	COMT
RoP02G04759	RoP05G01896	CCR	RoP06G01761	RoP06G03093	4CL
RoP02G03919	RoP07G00712	PAL	RoP06G01765	RoP06G03090	4CL
RoP02G05007	RoP08G00805	COMT	RoP06G02092	RoP11G00022	HCT
RoP02G02518	RoP08G01246	CCR	RoP07G00031	RoP08G00426	4CL
RoP02G00781	RoP10G01567	HCT	RoP07G00051	RoP08G00387	HCT
RoP02G00785	RoP10G01569	HCT	RoP07G00073	RoP08G00357	CCR
RoP02G02980	RoP10G02074	HCT	RoP09G00380	RoP09G00429	COMT
RoP02G00776	RoP10G01614	HCT	RoP01G05536	RoP10G00059	NAC
RoP04G03186	RoP07G02385	NAC	RoP01G00782	RoP03G04345	NAC
RoP01G01199	RoP09G02314	NAC	RoP04G02048	RoP07G00568	NAC
RoP01G05098	RoP11G01496	NAC	RoP01G01029	RoP09G02157	NAC
RoP01G04082	RoP03G04139	NAC	RoP01G05088	RoP11G01517	NAC
RoP04G01300	RoP07G01515	NAC	RoP02G05017	RoP08G00793	NAC
RoP01G05134	RoP11G01458	NAC	RoP06G02010	RoP11G00564	NAC
RoP06G02365	RoP11G00220	NAC	RoP04G01669	RoP07G01142	NAC
RoP04G01302	RoP07G01517	NAC	RoP01G03649	RoP03G03725	NAC
RoP01G03773	RoP03G03835	NAC	RoP03G01544	RoP06G02861	NAC
RoP01G03172	RoP09G01236	NAC	RoP01G03142	RoP09G01191	NAC
