# Supplementary material for: Genome and transcriptomics provide insights on stipular spine morphogenesis in Robinia pseudoacacia
Source: For Res (Fayettev). 2026 Jan 31;6:e003. doi: 10.48130/forres-0026-0003 (PMC13187913; doi:10.48130/forres-0026-0003)
Supplement: Supplementary file 1 — Supplementary data to this article can be found online. [file forres-6-1-e003-Supplementary.zip › 10.48130_forres-0026-0003-Suppl-TableS6.pdf]

Table S6. Quantification of lignin fractions by quantitative 2D-HSQC NMR method (results expressed per 100 Ar).

Samples	β-*O*-4	β-β	β-5	S/G
AGT-Sx	64.06	11.27	1.25	2.46
AGT-Ss	82.31	-	0.59	2.82
LC110-Sx	60.52	10.96	0.88	2.74
LC110-Ss	55.43	-	-	2.34
